# Case Report: Unmasking a sporadic pediatric tumor emergency: superior vena cava syndrome

**DOI:** 10.3389/fped.2025.1517745

**Published:** 2025-03-07

**Authors:** Gui-Liang Liu, Min Wang, Min Zhang, Yan Dai, Di-Wen Zhang

**Affiliations:** Department of Pediatrics, The People’s Hospital of Guangxi Zhuang Autonomous Region, Nanning, China

**Keywords:** superior vena cava syndrome, pediatric oncology, corticosteroid therapy, pointof-care ultrasound, multidisciplinary care, dyspnea, T-cell lymphoblastic lymphoma

## Abstract

**Introduction:**

Superior Vena Cava Syndrome (SVCS) is a rare but serious oncologic emergency in pediatric patients, most commonly caused by mediastinal masses such as lymphomas or leukemias. This condition results from the obstruction of the superior vena cava (SVC), leading to impaired venous return and respiratory and cardiovascular complications, progressive exacerbation in a short period, and an extremely high fatality rate. We report the case of a 12-year-old boy with SVCS caused by a mediastinal mass.

**Main symptoms/findings:**

The patient presented with progressive dyspnea, orthopnea, and swelling of the head and neck. He also exhibited chest tightness, dry cough, and shortness of breath. A chest CT revealed a large anterior mediastinal mass compressing the SVC and main bronchi.

**Diagnosis, treatment, outcomes:**

The patient was diagnosed with SVCS secondary to T-cell lymphoblastic lymphoma. Treatment began immediately with oxygen therapy and intravenous dexamethasone to reduce mediastinal compression. Significant clinical improvement was observed within 48 h, with a reduction in dyspnea and swelling. A biopsy confirmed T-cell lymphoblastic lymphoma and multidisciplinary care was pivotal to successful management.

**Conclusion:**

Early recognition and treatment of pediatric SVCS are essential to prevent life-threatening complications. Combined with a multidisciplinary approach, corticosteroid therapy was crucial for the patient's rapid recovery. Further research is needed to optimize treatment protocols and improve outcomes for pediatric SVCS cases.

## Introduction

SVCS is a rare but critical condition in pediatric patients (1), commonly caused by mediastinal masses such as lymphomas or leukemias. It occurs due to obstruction of the SVC, impairing venous return from the upper body and leading to significant cardiovascular and respiratory complications (2). Clinical manifestations include facial, neck, and upper limb swelling and respiratory distress.

In adults, SVCS is often associated with solid malignancies like lung cancer, lymphoma, and metastatic tumors. Conversely, in pediatric patients, SVCS is predominantly linked to hematologic malignancies such as non-Hodgkin lymphoma, acute lymphoblastic leukemia (ALL), and, less commonly, Hodgkin lymphoma. These conditions typically present with large mediastinal masses that compress vital structures, including the SVC, trachea, and bronchi (3).

While the majority of pediatric SVCS cases are oncological, the syndrome can occasionally result from benign conditions such as thrombosis, infections, or congenital heart abnormalities (4). Despite this, the rarity of SVCS in pediatric populations presents unique diagnostic and therapeutic challenges, often necessitating urgent medical attention due to the rapid onset and progression of symptoms (5). Treatment planning should be multidisciplinary (6), typically involving administering corticosteroids, such as dexamethasone, to reduce the size of the mediastinal mass and alleviate compression of the SVC, although it may complicate histopathological diagnosis (7).

The rapid onset of SVCS symptoms necessitates prompt diagnosis. Imaging, particularly computed tomography (CT), is critical for confirming the diagnosis and evaluating the mass's impact on surrounding structures. Point-of-care ultrasound (POCUS), performed by clinicians familiar with the patient's clinical status, offers a rapid bedside diagnostic tool that supports timely decision-making. Limited data exists in the literature on the use of POCUS in SVCS, with only a handful of case reports documenting its application in emergency settings (8).

In our patient, POCUS identified a vascular neck mass compressing the SVC, contributing to right heart dysfunction. Upright positioning alleviated pressure on the pulmonary vessel and trachea, improving respiratory and hemodynamic stability.

This case underscores the importance of rapid diagnosis and multidisciplinary management in pediatric SVCS, particularly in the context of oncological emergencies. Integrating advanced imaging and clinical expertise is essential in optimizing outcomes for these critically ill patients.

## Case presentation

### Patient information

**Figure 1:**
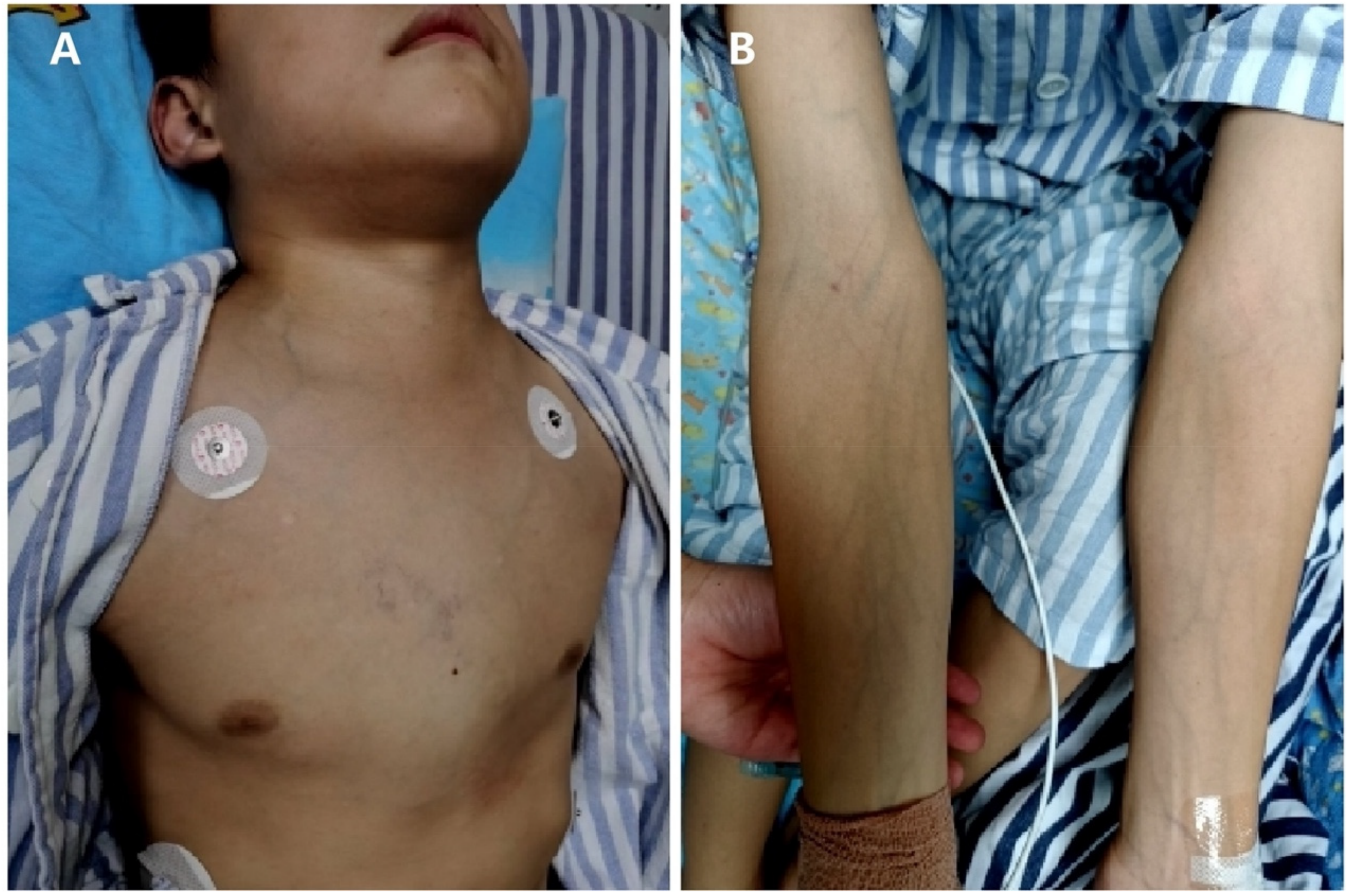
Physical signs of SVCS. **(A)** Swelling in the patient's head and neck. **(B)** Visible veins in the bilateral upper limbs.

A 12-year-old boy was admitted to the pediatric intensive care unit (PICU) due to worsening dyspnea and orthopnea. He had developed progressive swelling in his head and neck (Figure 1A) over the past month, accompanied by a dry cough, chest tightness, and shortness of breath in the preceding five days. His medical history was unremarkable.

### Clinical findings

On admission, his vital signs were: temperature 36.7°C, respiratory rate 24 breaths/min, pulse 120 beats/min, blood pressure 136/84 mmHg. He was alert and responsive but had difficulty speaking in complete sentences and preferred an upright position (orthopnea). His oxygen saturation was 92% on room air, improving to 98% with oxygen therapy via nasal cannula at 3 L/min. Physical examination revealed significant swelling in the head and neck, with visible distension of the jugular veins and veins in the bilateral upper limbs (Figure 1B) and anterior chest wall (Figure 1A); a large fixed mass was apparent over the right clavicle. Lung auscultation revealed asymmetric breath sounds with diminished sounds in the right lung, but no wheezing was noted.

### Diagnostic assessment

**Figure 2:**
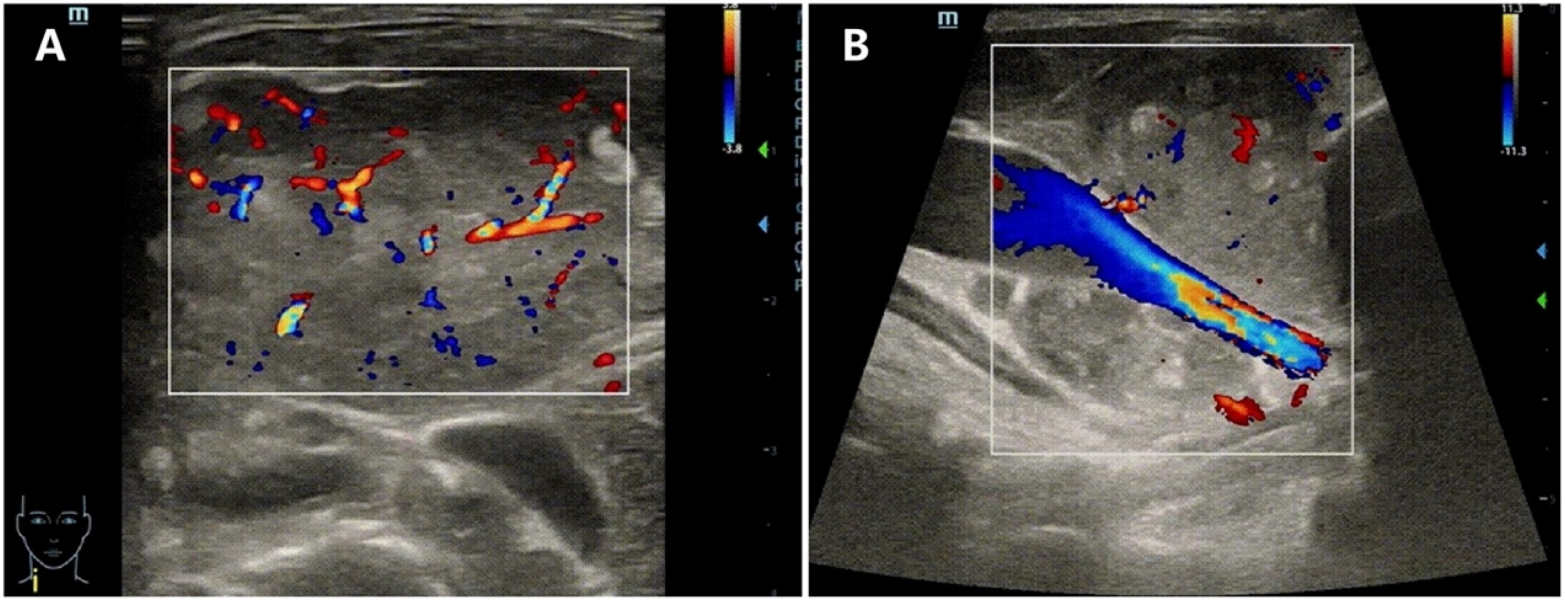
POCUS of the mass on the right clavicle. **(A)** The mass with abundant blood supply. **(B)** Accelerated blood flow at the site of narrowing of the SVC.

**Figure 3:**
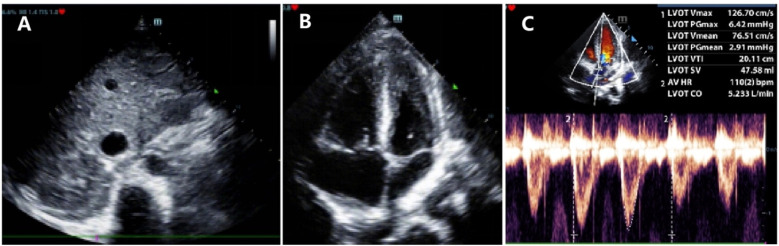
Point-of-care ultrasound of the heart. **(A)** Dilation of the inferior vena cava. **(B)** Right heart enlargement with increased wall motion. **(C)** Left Ventricular Output of 5.2 L/min.

SVCS is a condition caused by the obstruction of the superior vena cava, leading to symptoms such as facial swelling, neck vein distention, and shortness of breath. It is diagnosed through clinical symptoms, imaging studies (such as CT or chest x-ray), and sometimes biopsy to identify the underlying cause, often a malignancy. In our patient, a PICU physician used POCUS—guided by clinical questions at the bedside—to quickly and accurately assess the neck mass upon admission, evaluating its impact on the child's respiratory and circulatory systems. The POCUS indicated that the mass had abundant blood supply (Figure 2A), accelerated blood flow at the site of narrowing of the SVC (Figure 2B), wrapping around and compressing the SVC, suggesting an invasion of the vessel. Moreover, POCUS revealed dilation of the inferior vena cava (Figure 3A) with a respiratory variation rate of less than 50%, accompanied by right heart enlargement (Figure 3B) and hyperkinetic ventricular wall motion, suggesting that the mediastinal mass may be compressing the pulmonary vessels, thereby increasing right ventricular afterload and leading to right heart dysfunction. However, the patient maintained a left heart output of 5.2 L/min (Figure 3C) through forceful breathing and an elevated heart rate, though this compensation is fragile and limited.

**Figure 4:**
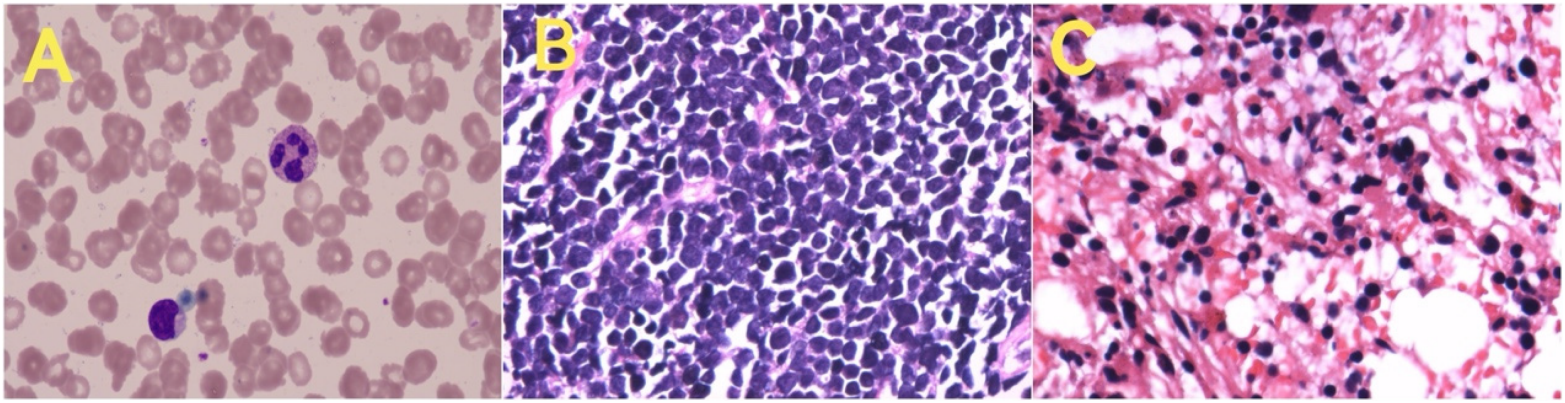
Smears from various tissues revealed the following findings: **(A)** Peripheral blood smear: generally normal, with no tumor cells observed. **(B)** Pathology of the right neck mass: consistent with T-cell lymphoblastic lymphoma. **(C)** Bone marrow pathology (left posterior superior iliac spine): findings consistent with T-cell lymphoblastic lymphoma involving the bone marrow.

**Figure 5:**
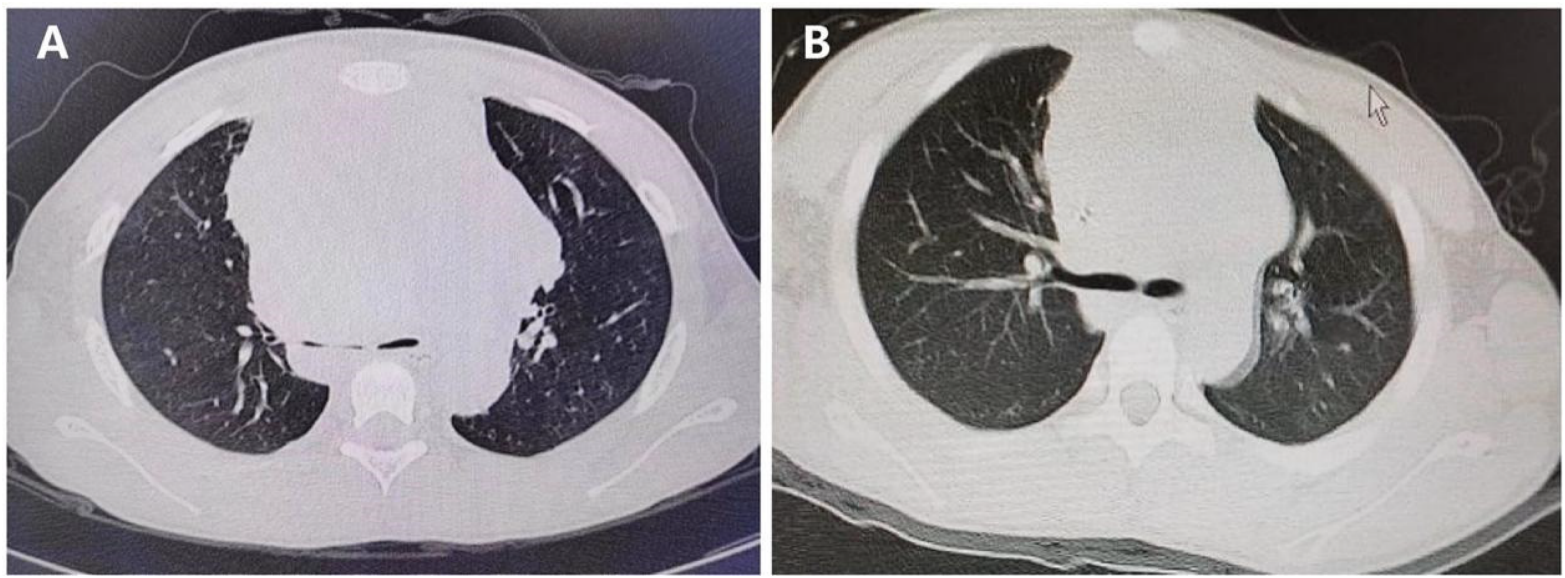
Lung CT before and after steroid Use. **(A)** Severe compression of the main bronchi before steroid use. **(B)** Reduction in bronchial compression after seven days of steroid therapy.

Blood tests, including a complete blood count, lactate dehydrogenase, and tumor markers, showed no significant abnormalities (Table 1). Peripheral blood cell morphology showed no evidence of tumor cells (Figure 4A). A chest CT scan performed one hour after admission showed a mass in the anterior superior mediastinum and right supraclavicular region, with severe compression of both main bronchi, nearly obstructing the right side completely (Figure 5A). Multiple enlarged lymph nodes were also noted, indicating a likely diagnosis of anterior mediastinal malignancy with lymph node metastasis. Electrocardiography revealed sinus tachycardia, and an echocardiogram confirmed a hyperdynamic left heart that compensated for the compromised right heart function due to the mass effect.

**Table 1:** Blood Gas analysis, complete blood count, infection markers, and liver and renal function within 48 hours of admission.

Blood tests	Time after admission	Body position	PH	PO_2_ (mmHg)	PCO_2_ (mmHg)	PO_2_/FiO_2_	Lac (mmol/L)
Blood gas analysis	1 h ost-admission	Supine position	7.37	51.3	41.5	177	1.2
	4 h post-admission	Seated position	7.33	88.8	42.5	306	1.1
	10 h post-admission	Semi-lateral position	7.31	101	41.4	347	1.4
	24 h post-admission	Semi-lateral position	7.42	153	45.1	376	0.8
	48 h post-admission	Supine position	7.45	112	36.7	387	1.1
Complete blood count CRP, PCT	WBC (10^9^/L)	*N* %	L%	HB (g/L)	PLT (10^9^/L)	CRP (mg/L)	PCT (ng/ml)
	4.6	55.5	30.4	101	214	10	0.11
Liver and renal function	Tbil (umol/L)	ALB (g/L)	ALT (U/L)	AST (U/L)	Creatinine (umol/L)	Urea (mmol/L)	LDH (U/L)
	7.6	45.5	8	26	61	5.4	1,007

### Treatment intervention

Given the critical nature of the patient's condition, immediate treatment with intravenous dexamethasone (0.11 mg/kg/day, for 3 consecutive days) was initiated to reduce the mass effect and alleviate SVCS. Due to the high risk of tumor lysis syndrome, consultation with a pediatric hematologist-oncologist was conducted, and preventive measures were implemented.

A multidisciplinary team (MDT), including specialists from PICU, Pediatric Hematology-Oncology, Anesthesia, Ultrasound, and Pediatric Surgery, was convened within eight hours of admission. Under local anesthesia and with PICU support, a percutaneous core needle biopsy of the right supraclavicular mass and bone marrow puncture was performed 12 h after admission; both biopsies later confirmed T-cell lymphoblastic lymphoma with bone marrow involvement (Figures 4B,C).

### Clinical outcome

**Figure 6:**
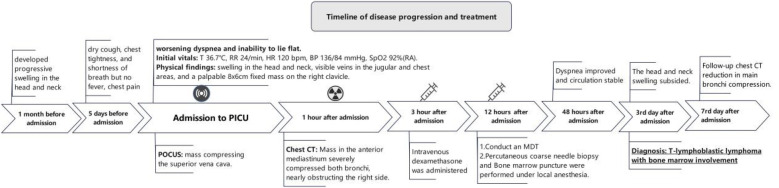
A timeline of symptom onset, clinical management, and treatment response in a patient with lymphoblastic lymphoma and mediastinal compression leading to respiratory distress.

The patient showed significant clinical improvement within 48 h of admission, with resolution of dyspnea and circulatory stabilization. Swelling in the head and neck subsided by the third day, and a follow-up lung CT scan on the seventh day revealed reduced compression of the main bronchi (Figure 5B). A comprehensive timeline of symptom onset, clinical management, and treatment response highlights the effective management of lymphoblastic lymphoma with mediastinal compression leading to respiratory distress (Figure 6).

## Discussion

SVCS is a rare but critical condition in pediatric patients, often signaling the presence of a mediastinal mass, such as lymphoma or leukemia. In children, it is considered an oncologic emergency (9) because it can lead to cardiac and pulmonary dysfunction and potentially resulting in cardiopulmonary failure and death. The primary causes of SVCS in children are mediastinal mass, central venous catheter-related thrombosis, and complications related to congenital heart disease treatment. The most common malignant tumors causing SVCS in the anterior mediastinum are non-Hodgkin lymphoma, leukemia, and Hodgkin lymphoma (10). Furthermore, clinical manifestations result from the tumor's compressive effect, such as SVCS, dyspnea, cough, orthopnoea, dysphagia, hoarseness, anxiety, confusion, and syncope. In our patient, the patient presented with classic signs of SVCS, including head and neck swelling, visible chest veins, and respiratory distress. The presence of these symptoms, combined with imaging findings, led to the rapid diagnosis of an anterior mediastinal mass, which presents as a complex oncologic emergency that demands immediate and multifaceted attention in accordance with established management principles. Critical considerations include stabilizing the patient's condition and utilizing minimally invasive diagnostic procedures (11). In moderate to high-risk cases, the use of general anesthesia should be minimized, with a preference for local anesthesia for tissue biopsy. If general anesthesia is unavoidable, a comprehensive plan that considers spontaneous breathing and dynamic blood pressure monitoring should be implemented (12). The management of SVCS in pediatric patients presents unique challenges due to the rapid onset and potential severity of symptoms. The presence of a mediastinal mass remains one of the most concerning features in the assessment of a child referred for anesthesia or sedation because of the potential for life-threatening events under anesthesia (13). General anesthesia can occur a significant airway obstruction in a diagnosed case of anterior mediastinal mass (14). Improper interventions, such as sedation, muscle relaxants, positive pressure ventilation, and general anesthesia (15), can lead to rapid cardiac and pulmonary failure and even death. Qureshi et al. (16) reported a case of a 6-year-old child who was misdiagnosed with foreign body aspiration, later diagnosed with SVCS caused by T-cell acute lymphoblastic leukemia. The child underwent two instances of general anesthesia for bronchoscopy and mass biopsy, both of which resulted in sudden hypoxic episodes post-anesthesia. The first episode even led to cardiac arrest, necessitating cardiopulmonary resuscitation and the termination of the procedures.

The decision to initiate corticosteroid therapy early in our patient was driven by the urgent need to reduce the mediastinal mass and alleviate its severe compression on the airway. In pediatric patients with mediastinal masses, lymphoma is the most common cause of SVCS, making early corticosteroid use clinically appropriate for life-saving purposes. However, it is important to note that corticosteroids may be ineffective for other tumor types, such as acute myeloid leukemia with mediastinal granulocytic sarcoma, which does not respond to this treatment (17).

However, the use of steroids prior to obtaining a biopsy carries the risk of altering the histopathological features of the tumor, potentially complicating the diagnosis (18). Following the administration of corticosteroids, it is crucial to perform a biopsy of the mass as soon as possible and promptly adjust the chemotherapy regimen based on the pathological findings. This approach ensures rapid tumor reduction and alleviation of respiratory and circulatory instability caused by mass compression. In our patient, corticosteroid treatment led to a rapid reduction in the mass size, effectively relieving the compressive effects. Within 12 h of admission, the patient was diagnosed with T-cell lymphoblastic lymphoma, a result achieved through the close collaboration of the multidisciplinary team (MDT).

The role POCUS was pivotal in our patient, providing real-time assessment of the hemodynamic impact of the mediastinal mass (19). POCUS revealed right heart enlargement, ventricular septal deviation, and elevated central venous pressure, all indicative of significant cardiovascular compromise. This information was crucial in guiding the management plan, including avoiding excessive fluid administration and sedation, which could have precipitated circulatory collapse. In severe cases, an ECMO team should be readily available to manage sudden life-threatening respiratory and circulatory failure (20).

The case also highlights the critical importance of early recognition and intervention in SVCS. Delays in diagnosis or treatment can lead to catastrophic outcomes, including sudden cardiac and respiratory failure. The rapid improvement in the patient's symptoms following corticosteroid therapy and the reduction in bronchial compression observed on follow-up CT scans demonstrate the efficacy of timely intervention in preventing severe complications.

Despite the successful outcome in our patient, several challenges remain in the management of SVCS in pediatric patients. The rarity of the condition means that many pediatricians may have limited experience in recognizing and treating it. Additionally, the optimal timing and dosing of corticosteroids, as well as the potential need for additional therapies such as chemotherapy or radiation, require further study. The long-term prognosis for patients with SVCS secondary to lymphoma also remains an area of ongoing research, particularly in understanding the factors that contribute to relapse or recurrence.

## Conclusions

This case underscores the critical need for early recognition and management of SVCS in pediatric patients with mediastinal masses. The successful outcome was largely due to the timely intervention by a multidisciplinary team, the strategic use of steroids, and the application of POCUS in the PICU setting. Further studies are warranted to refine the management protocols for SVCS in pediatric oncology.

## Data Availability

The original contributions presented in the study are included in the article/Supplementary Material, further inquiries can be directed to the corresponding author.
